# Detection of Adulterants in Apple Juice Concentrate by Physicochemical Properties, Organic Acids Profile, Minerals, and Multivariate Classification Strategies

**DOI:** 10.1002/fsn3.72035

**Published:** 2026-06-16

**Authors:** Samal Yeganeh‐Zare, Khalil Farhadi, Saber Amiri

**Affiliations:** ^1^ Department of Analytical Chemistry, Faculty of Chemistry Urmia University Urmia Iran; ^2^ Institute of Nanotechnology Urmia University Urmia Iran; ^3^ Department of Food Science and Technology, Faculty of Agriculture Urmia University Urmia Iran

**Keywords:** adulteration, apple juice concentrate, mineral, multivariate statistical analysis, organic acids profile, physicochemical properties

## Abstract

From a food safety perspective, with a focus on natural fruit juices and concentrates, various types of adulteration, such as substitution with cheaper sweeteners, are increasingly recognized. In the present work, pure apple juice concentrate was adulterated with fructose syrup, glucose syrup, and date concentrate. Physicochemical properties (acidity, reducing sugars, formalin index, Brix, and total sugar), organic acid profiles (lactic acid, ascorbic acid, fumaric acid, and malic acid), and minerals (magnesium, iron, zinc, copper, sodium, and potassium) of pure and adulterated apple juice concentrate were evaluated and used to detect adulteration. For this purpose, after reducing the number of parameters determined by principal component analysis (PCA) and identifying the principal components (PCs) using linear and quadratic discriminant analyses (LDA and QDA), 15 parameters (including 4 organic acid factors, 5 physicochemical factors, and 6 mineral elements) were applied to classify the adulteration and authenticity of apple juice concentrate. Based on the results, the first four PCs that showed eigenvalues greater than 1 accounted for 84.5% of the total variance of the data set. The best classification (100%) was obtained using the values of three mineral elements, including sodium, potassium, and copper. Also, two physicochemical parameters, pH and total sugar, as well as the amounts of two organic acids, malic acid and ascorbic acid, were identified as the best parameters for classifying the type and concentration of adulterations in apple concentrate, respectively. The obtained results illustrated supreme discrimination for the type and concentration of the adulterants with a zero 2Log (likelihood) factor and 100% correct classification, which can be used for reliable detection of adulteration in apple concentrate.

## Graphical abstract

This study presents a new method for detecting date syrup, glucose, and fructose adulteration in apple juice concentrate. The approach combines physicochemical property and minerals measurements as well as HPLC profiling of organic acids with chemometric analysis.

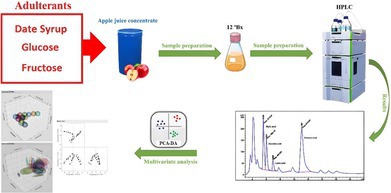

## 1 Introduction

The fruit juice industry is one of the largest agribusinesses. Fruit juices and concentrates benefit humans by being a primary source of nutrients and energy and by maintaining human health. Several natural products such as pomegranate, apple, peach, and others are utilized in juice production. Due to the diverse nutrient content of apples and their potential to prevent several diseases such as cancer, obesity, and diabetes, the use of apple juice has increased compared to other fruits (Hyson 2011; Hodgson et al. 2016). For this reason, the possibility of using cheaper natural fruit juices, pulp, or other sugar sources like fructose‐rich corn syrup, hydrolyzed inulin syrup, inverted syrup, cane, and beet sucrose as adulterants in the apple juice industry has greatly increased (Thavarajah and Low 2006; Amiry et al. 2017; Miaw, Sena, de Souza, Ruisanchez, and Callao 2018; Dasenaki and ThomaidisKelly 2019).

Apple juice contains organic acids, phenolic compounds, sugars, volatile organic compounds, macro‐ and micro minerals including magnesium (Mg), potassium (K), and calcium (Ca), as well as iron (Fe), zinc (Zn), copper (Cu), selenium (Se), and boron (B), which can be used to determine quality (Spinelli et al. 2016; Jan et al. 2016; Pandurang et al. 2020; Cvetković et al. 2024) and distinguish between natural and adulterated products (Kelebek et al. 2009).

On the other hand, the date fruit contains a wide range of functional nutritional components. It is rich in easily digestible sugars such as glucose and fructose. It represents a good source of fiber and trace elements such as potassium, phosphorus, magnesium, calcium, selenium, and iron, and vitamins such as ascorbic acid, niacin, and pyridoxine. It also contains bioactive components such as anthocyanins, phenolics, carotenoids, procyanidins, and flavonoids, so date concentrate can be a suitable object for adulteration of apple juice because of being cheap and having more similarities in functional nutritional components with that (Bouhlali et al. 2020; Allaith 2008). Moreover, as Iranian apple juices are exported to many countries worldwide, it is essential to screen their authenticity and quality by measuring their chemical composition, physicochemical attributes, minerals, organic acids, and other parameters. According to our knowledge, there is one report in the literature about adulteration of Iranian apple juice concentrate with date concentrate and glucose or fructose syrup (Yeganeh‐Zare et al. 2021).

In general, numerous analytical indicators can be analyzed for a large amount of juice, creating a complex data set. High‐performance liquid chromatography (HPLC) is one of the most reliable and well‐defined instruments for analyzing specific compounds. Currently, measurement of carbohydrate profiles with HPLC‐RID is one of the most commonly used official, susceptible, and authentic methods for analyzing apple juice purity (Yeganeh‐Zare et al. 2021). Therefore, the application of alternative methods by high‐performance tests as minerals, organic acids, or physicochemical properties can be another appropriate way to determine adulterations. Chemometrics is an interdisciplinary science that uses computational, numerical, and statistical techniques to extract data from chemical systems through mathematical modeling. The current discriminant analysis (DA) and principal component analysis (PCA) applications allow for the review and categorization of accumulated information from different samples. DA is closely associated with PCA as both techniques look for linear combinations of variables that best describe the data. DA tries to model the differences between the different statistical categories. This approach is one of the most observed modeling approaches widely used in linear discriminant analysis (Amiry et al. 2017; Fahim et al. 2022).

The study combines multiple analytical techniques (physicochemical properties, organic acids profile, minerals) with multivariate classification strategies, providing a holistic approach to detecting adulterants. The use of advanced multivariate classification strategies adds a modern and innovative dimension to the study, enhancing the accuracy and reliability of adulterant detection. The exploration of alternative methods alongside HPLC for analyzing apple juice purity showcases the study's forward‐thinking approach to food analysis. The findings can have significant practical implications for food safety and quality control in the juice industry, contributing to improved standards and practices. The study's interdisciplinary nature, integrating chemistry, food science, and data analysis, provides valuable insights that can inspire further research and development in related fields. Therefore, the objectives of this review are to distinguish adulterated apple juices and to classify these juices according to physicochemical, mineral, and organic acids information using chemometrics methods.

## 2 Methods and Materials

### 2.1 Chemicals and Reagents

Extra pure sulfuric acid, sodium hydroxide 0.1 N, formaldehyde, and phenolphthalein were bought from Merck (Darmstadt, Germany), and pure water was prepared with Milli‐Q water (Millipore Corp., Saint‐Quentin, France). L‐malic, L‐ascorbic, L‐lactic, and L‐fumaric acids were also obtained from Sigma‐Aldrich (Aldrich, Steinheim, Germany) with high purity. Standard solutions containing Mg, Fe, Zn, Cu, Na, and K at a concentration of 1000 ppm were purchased from Merck (Darmstadt, Germany). All reagents and standard solutions were filtered through a 0.45‐μm pore‐size membrane filter before use.

### 2.2 Materials

Pure and authenticated apple juice concentrate (PAJC) was bought directly from Shadli Co. (Urmia, West Azerbaijan Province, Iran). Date concentrate (DC) was purchased from Sansanshahd Co. (Urmia, West Azerbaijan Province, Iran). Fructose and glucose syrup (FS & GS) were bought from Zarnam Co. (Tehran, Iran).

### 2.3 Preparation of Adulterated Apple Juice Concentrate ( **AAJC** ) Samples

Pure apple juice concentrate was transferred to a 50 mL falcon and stored at 4°C ± 1°C to preserve physicochemical properties, minerals, and organic acid profiles. PAJC was mixed with DC, FS, and GS to prepare adulterated samples, which covered a wide range in concentration from 90% apple juice concentrate with 10% of the adulterant to 50% apple juice concentrate and 50% of the adulterant, with 4 replications for each batch (total samples = 60, Figure S1). Due to the lack of economic benefit, impurity of less than 10% was not studied. All samples were analyzed at Brix 12.

### 2.4 Analysis of Physicochemical Properties

#### 2.4.1 **pH** Assay

The pH of apple juice samples was measured at 25°C ± 1°C using a Metrohm 827 pH meter (Metrohm AG, Switzerland) after calibration with pH buffer solutions 4 and 7 (AOAC, 2005).

#### 2.4.2 Free Acidity Determination

Measuring free acidity was carried out with titration of a 10 g apple juice sample by NaOH solution (0.1 M) in the presence of phenolphthalein and represented as malic acid per 100 g of apple juice (AOAC, 2005).

#### 2.4.3 Reducing Sugar and Total Sugar Determination

The reducing sugar (g/100 g of juice) in the apple juices was specified by titration with Fehling's solution and using methylene blue as an indicator. Total sugar (g/100 g of juice) was also determined in the same way, after the reduction of sucrose by hydrochloric acid.

#### 2.4.4 Formalin Index

The procedure for determining the formalin index in apple juices consisted of neutralizing the apple juice sample with NaOH solution (1 N) to give a pH of 8.1, then adding an excess of neutralized formaldehyde, and re‐titrating the resulting solution with NaOH solution (0.1 N) to pH 8.1 as the endpoint (AOAC, 2005).

### 2.5 Organic Acids and Chromatographic Analysis

For the determination of organic acids, the samples were diluted with sulfuric acid (pH = 2.5), passed through 0.45 μm membrane filters, and analyzed by HPLC on an Agilent 1260 series with a Zorbax‐SBC18 column (250 × 4.60 mm, 5 μm; Agilent, USA) at an oven temperature of 40°C. The mobile phase was H2SO4 0.002 N (pH 2.5) in isocratic mode with a flow rate of 0.65 mL/min and an injection volume of 20 μL of the sample. L‐ascorbic, L‐malic, L‐fumaric, and L‐lactic acids were identified by a DAD detector at 215 nm. Data processing was carried out with the Agilent Chemstation software. This method was presented by Andrés et al. (2014). The chromatogram of each organic acid was identified and quantified by comparing the retention time and the peak area with the external standard.

### 2.6 Mineral Composition

The mineral composition was determined using the method described by Bouhlali et al. (2017). Briefly, two grams of each sample were placed in a previously weighed porcelain crucible and heated in a furnace at 550°C until constant mass. The resulting ashes were dissolved in five milliliters of concentrated hydrochloric acid, and the mixtures were heated on a hot plate until complete dryness. Then, 5 mL of deionized water was added and made up to 25 mL in a calibrated flask. The resulting solutions were used for mineral content determination. Atomic absorption spectrometry (240FSAA, Varian, USA) was implemented to determine Fe, Mg, Cu, Zn, K, and Na levels.

### 2.7 Chemometrics Analysis

In this study, the results of the organic acids profile (L‐malic, L‐ascorbic, L‐lactic, and L‐fumaric acids) by HPLC‐DAD, physicochemical properties (pH, free acidity, reducing sugar, total sugar, and formalin index), and minerals (Mg, Fe, Zn, Cu, Na, and K) were applied to chemometric analysis. For this aim, principal component analysis (PCA) and linear discriminant analysis (LDA) were used to discriminate samples according to the concentrations and types of adulterants. PCA was performed to reduce the data dimensions using eigenvalue methods to obtain a data component set based on the correlation of original parameters with 95% confidence. LDA was performed to establish the discrimination model relating to the organic acids profile, minerals, and physicochemical properties data of the samples. In this work, the amount of each organic acid, mineral, and physicochemical property was quantified for each adulterated sample. The multivariate analysis and plotting were done using JMP software version 16 (SAS Institute, Cary, USA) (Bari et al. 2021).

## 3 Results and Discussion

### 3.1 Physicochemical Properties, Minerals, and Organic Acids Profile of **AAJC**

The results for pH, titratable acidity, formalin index, reducing sugar, total sugar, malic acid, ascorbic acid, lactic acid, fumaric acid, Mg, Fe, Zn, Cu, Na, and K of the apple juice concentrate, date concentrate, fructose, and glucose syrup in Brix 12 are given in Table 1. Significant differences were revealed among the studied samples for the analyzed factors.

**TABLE 1:** Physicochemical properties, organic acids profile, and minerals in experimental apple juice concentrate, glucose syrup, fructose syrup, and date concentrate (Brix = 12).

Analysis factors	Apple juice concentrate^a^			Glucose syrup^b^			Fructose syrup^c^			Date concentrate^d^		
	Min.	Max.	Mean ± SD	Min.	Max.	Mean ± SD	Min.	Max.	Mean ± SD	Min.	Max.	Mean ± SD
**pH**	3.96	4.06	4.01 ± 0.07	4.85	4.99	4.92 ± 0.1	4.17	4.21	4.19 ± 0.03	4.15	4.59	4.37 ± 0.31
**Titrable Acidity**	0.23	0.24	0.235 ± 0.01	0.01	0.01	0.01	0.01	0.01	0.01	0.17	0.19	0.18 ± 0.02
**Formalin Index**	3	3.5	3.25 ± 0.35	N. D.^e^	N.D	N.D	N.D	N.D	N.D	8.4	6.88	7.64 ± 1.07
**Reducing Sugar**	8.59	8.61	8.6 ± 0.01	3.59	3.65	3.62 ± 0.04	11.51	12.3	11.91 ± 0.55	9.51	9.44	9.48 ± 0.05
**Total Sugar**	10.72	10.81	10.76 ± 0.06	3.61	3.67	3.64 ± 0.04	11.51	12.3	11.91 ± 0.55	9.64	9.58	9.61 ± 0.04
**Malic acid**	2.6	3.04	2.89 ± 0.14	N.D	N.D	N.D	N.D	N.D	N.D	0.24	0.28	0.26 ± 0.03
**Ascorbic acid**	0.038	0.06	0.054 ± 0.006	N.D	N.D	N.D	N.D	N.D	N.D	1.41	1.43	1.42 ± 0.01
**Lactic acid**	0.25	0.42	0.26 ± 0.1	N.D	N.D	N.D	N.D	N.D	N.D	0.745	0.9	0.82 ± 0.11
**Fumaric acid**	0.002	0.004	0.003 ± 0.0001	N.D	N.D	N.D	N.D	N.D	N.D	7.87	8.9	8.38 ± 0.72
**Mg (ppm)**	35.47	37.30	36.38 ± 1.30	14.53	14.79	14.68 ± 0.12	0.68	0.79	0.73 ± 0.05	43.16	104.6	73.61 ± 23
**Zn (ppm)**	0.55	0.59	0.57 ± 0.03	0.52	0.56	0.54 ± 0.02	0.51	0.73	0.61 ± 0.11	0.74	1.07	0.91 ± 0.18
**Fe (ppm)**	5.62	6.01	5.81 ± 0.30	0.49	0.55	0.53 ± 0.03	0.40	0.48	0.44 ± 0.03	9.82	10.20	10.02 ± 0.16
**Cu (ppm)**	0.35	0.41	0.38 ± 0.04	0.45	0.48	0.46 ± 0.02	0.31	0.32	0.31 ± 0.01	2.66	3.01	2.83 ± 0.20
**Na (ppm)**	11.56	13.54	12.55 ± 1.40	80.13	85.89	83 ± 3.33	16.57	17.18	16.87 ± 0.35	18.27	20.18	19.23 ± 1.10
**K (ppm)**	977.00	988.00	982.00 ± 7.03	12.72	17.19	14.95 ± 2.58	24.32	27.74	26.03 ± 1.97	650.80	700.1	675.45 ± 28

*Note:* a–d, *p* < 0.05; e, not detected.

As shown in Table 1, the pH values ranged between 3.96 and 4.99 in PAJC, DC, FS, and GS. The pH value of the studied apple juice in the current research is in accordance with reported values by Liu et al. in 2018. The pH results in fructose and glucose syrup and date concentrate juice were higher than in apple juice.

Acidity is an important property of apple juice and is easily measured. Whether for making apple juice or blending fresh apple juice, it is always a good idea to measure acidity (Jan et al. 2016). Titratable acidity is another physicochemical property factor that can be effective in controlling the quality of apple juice (Zhang et al. 2018). According to Table 1, the titratable acidity ranged from 0.24 to 0.01 (g/100 g juice). The highest titratable acidity was in PAJC and the lowest in GS and FS. Therefore, the addition of FS, GS, and DC to PAJC reduced the titratable acidity.

Formalin index is a parameter used to evaluate the quality of fruit juice. This index is a measure of the amino acid content of fruit juice concentrate. According to Table 1, FS and GS contents have no formalin index, and DC has the highest formalin index. The formalin index of AAJC increases with increasing DC percentage and decreases with increasing FS and GS percentage. Examining the formalin index in apple juice concentrate is a suitable method to find its purity. On the other hand, the decrease in formalin index and titratable acidity can be used as indicators to distinguish AAJC with FS and GS. Also, AAJC with DC can be shown by increasing formalin index and decreasing titratable acidity.

Total and reducing sugars can be used as other markers for detecting adulterated apple juice concentrates. As observed in Table 1, fructose syrup has the highest amount of reducing sugar, and glucose syrup has the lowest amount. Results show that adulteration of fructose and glucose syrup in the apple juice concentrate has different effects on reducing sugar. A study of the total sugar amount and the ratio of total sugar to reducing sugar in apple juice concentrate can help us to find its adulteration with different syrups. Apple juice concentrate has the highest content of this ratio, and it is negligible in fructose syrup, glucose syrup, and date concentrate, so a decrease in total sugar to reducing sugar ratio indicates adulterated apple juice concentrate with fructose, glucose, and date concentrate.

**FIGURE 1:**
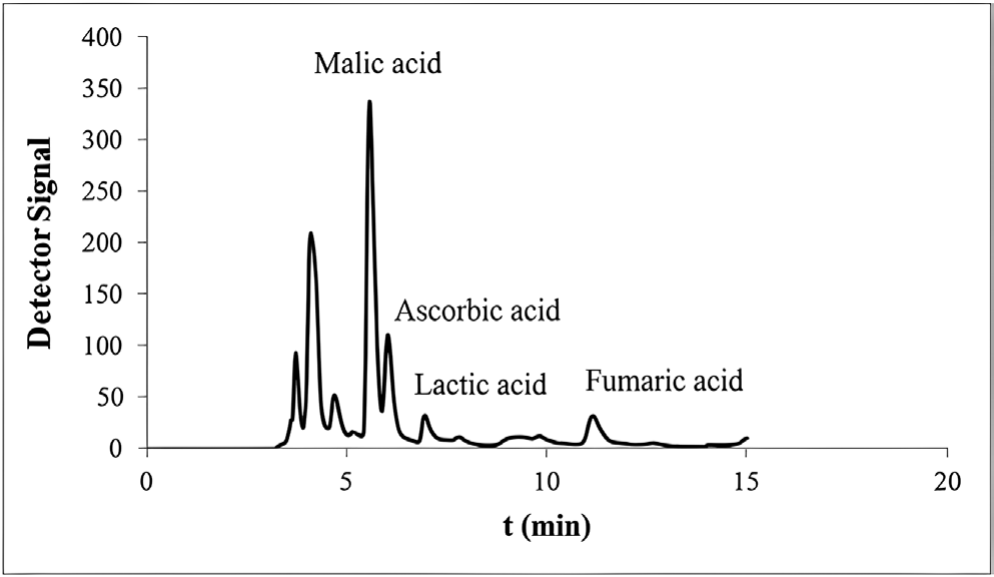
Chromatogram from HPLC‐DAD showing organic acids profile with corresponding retention times in natural apple juice concentrate.

The organic acid profiles were determined for PAJC (Figure 1) as well as for three common adulterants, including GS, FS, and DC. They were chosen based on the likelihood of use as adulterants in PAJC. Besides, they were selected due to their high similarity in carbohydrate composition compared to PAJC (Miaw, Sena, de Souza, Callao, and Ruisanchez 2018), which made them likely candidates for adulteration (Bouhlali et al. 2017, 2020).

In the analyzed apple juice concentrates, the organic acids profile is a significant component, and malic acid was the main organic acid (Mezey and Mezeyova 2018). The average malic acid content was 4.83 (g/l) (M et al. 2015), and according to the study by Wu et al. (2007), the average malic acid content was 4.61 (g/l); the highest content was 7.07 (g/l), and the lowest was 2.73 (g/l), while the malic acid content of experimental apple juice concentrate ranged from 2.6 to 3.04 (g/l) in Table 1. According to Table 1, GS and FS have no organic acids, so a reduction in the concentration of organic acids indicates the mixing of PAJC with GS and FS. The organic acids profile was determined for AAJC with 40% of GS (Supporting Information Figure S2).

According to Table 1 and reported results by Bouhlali et al. 2020, the amount of ascorbic acid, fumaric acid, and lactic acid in DC is noticeable compared to PAJC. On the other hand, DC has less malic acid than PAJC in Table 1. So, adding DC increases the amount of fumaric acid and ascorbic acid and decreases the amount of malic acid in PAJC. Therefore, fumaric and malic acids act as markers in AAJC with DC, and chromatograms from HPLC‐DAD for the organic acids profile in AAJC with 40% of DC confirm this (Supporting Information Figure S3).

In this research, to determine the quality of apple juice and identify its adulteration, the elements of Mg, Fe, Zn, Cu, Na, and K were investigated using the atomic absorption technique. For this purpose, the amount of these elements was measured in PAJC, DC, GS, and FS as suitable substances for adulteration. The results of this analysis are summarized in Table 1. According to the results of Table 1, PAJC has significant amounts of potassium and magnesium, which are very low in GS and FS. As noted, the amount of sodium in PAJC and FS is low, while it is significant in GS. Therefore, simultaneous examination of sodium, potassium, and magnesium concentration can be effective in identifying AAJC with GS and FS. By examining the copper and zinc concentration in PAJC, GS, and FS, no significant difference is observed. Compared to GS and FS, PAJC has a higher amount of iron, and iron content will be another indicator to identify the adulteration of PAJC with GS and FS. The obtained results from the analysis of the elements in DC in Table 1 indicate a high amount of Mg, Fe, and Cu compared to the amount of these elements in PAJC. PAJC has the highest amount of potassium compared to DC, GS, and FS. Adding DC to PAJC causes a very slight decrease in the amount of potassium, and analyzing the amount of potassium alone cannot indicate its adulteration with DC. Many studies have been conducted on mineral elements such as sodium, potassium, calcium, and magnesium in other fruit juices, and they have been used as adulteration indicators. In a study on orange juice, it has been shown that orange juice has high amounts of potassium and a decrease in potassium concentration is a sign of excessive dilution with water (Schmutzer et al. 2016). The same finding has been confirmed in the case of pomegranate juice, indicating that pomegranate juice containing potassium less than 2000 ppm is highly suspected of adulteration, likely from dilution with water or other fruit juices such as grape and peach juices (Nuncio‐Jauregui et al. 2014). In another study in 2010, it was found that if the ratio of potassium to magnesium is less than 50%, it can indicate an increase in sweetener in orange juice (Muntean 2010). A high concentration of sodium can indicate the addition of preservatives or adulterated ingredients (Schmutzer et al. 2016). However, determining the amount of sodium, potassium, calcium, and magnesium alone cannot be a suitable indicator for identifying adulteration in fruit juices. It is better to compare and check with other analysis factors.

In the first step of this work, after separating the organic acids by HPLC‐DAD, the physicochemical properties and minerals were studied. In the second step, similarities and differences between different adulterants and their levels of organic acids profiles, minerals, and physicochemical properties were statistically evaluated using multivariate techniques and statistical analysis.

### 3.2 **PCA** of **AAJC**

As mentioned, 60 adulterated samples of apple juice concentrate in 15 batches with 4 replications were considered to detect adulteration. The physicochemical properties, organic acids profile, and minerals, including pH, titratable acidity, formalin index, reducing sugar, total sugar, malic acid, ascorbic acid, lactic acid, fumaric acid, Mg, Fe, Zn, Cu, Na, and K, were determined for each sample. The most commonly utilized method for food adulteration analysis, which can reduce the dimensions of the data, is Principal Component Analysis (PCA) (Esteki et al. 2018). PCA is a multivariate procedure that provides a structural extraction technique based on a variance–covariance matrix or an association between dependent variables. The determination of the number of principal components by data group can be expected from the principal components (PCs) analysis (Fahim et al. 2022). As reported in recent references, the data of sugar and organic acid compounds gained from all juice samples were contained in PCA. The first two principal components accounted for 85.739% of the total variation by sugar amount as analytical data for PCA and, by organic acid content of this work, the first two principal components accounted for 59.767% of the total variation. The juice samples were classified into seven groups according to fruit types. Results displayed that all seven groups of fruit juice samples were separated distinctly in the score plots, showing a clear separation of these fruit juices according to type (Li et al. 2020). In our work, the first four PCs, which showed eigenvalues greater than 1, accounted for 84.5% of the total variance in the data group. The first four PCs' eigenvalues were 6.94, 2.72, 1.88, and 1.12. These 4 PCs were found to be the most informative for classifying samples in a three‐dimensional space. The ability of 4 PCs to discriminate adulteration according to the types and concentrations of adulterants was compared with the ability of all measured parameters. The differentiation rates, using leave‐one‐out cross‐validation, were used to optimize the number of PCs. The number of principal component variables determines the output of the LDA and Quadratic Discriminant Analysis (QDA) separation models.

**FIGURE 2:**
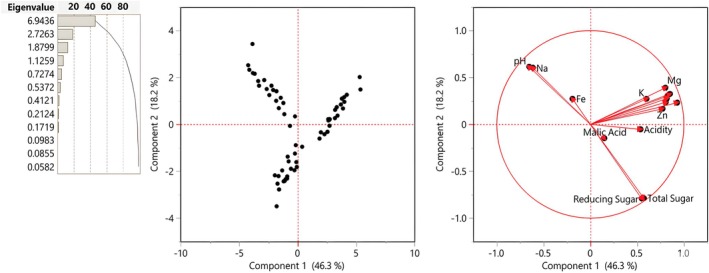
The biplot PCA results for 15 analysis factors of physicochemical properties, minerals, and organic acids profile in adulterated apple juice concentrate.

Figure 2 presents the results of the PCA analysis. The primary key module explained 46.3% and the subsequent 18.2% of the total variance.

### 3.3 Classification of AAJC by Linear Discriminant Analysis (LDA) and Quadratic Discriminant Analysis (QDA)

Linear Discriminant Analysis (LDA) and Quadratic Discriminant Analysis (QDA) were performed on z‐normalized results to analyze the class scores of samples according to adulteration sets. The likelihood factor is used to assess the importance of each canonical, which could be a comparison of how well each canonical segregates objects into sets. This factor is generally represented as −2 log (likelihood), and it shows the differentiation authority. Separation among sets was studied by plotting the first and second canonical and by applying a 95% certainty circle around the means (center of clusters) of each batch. In this work, we used four steps for discrimination analysis based on physicochemical parameters, minerals, organic acids profile, and their combination with each other.

#### 3.3.1 First Step

**FIGURE 3:**
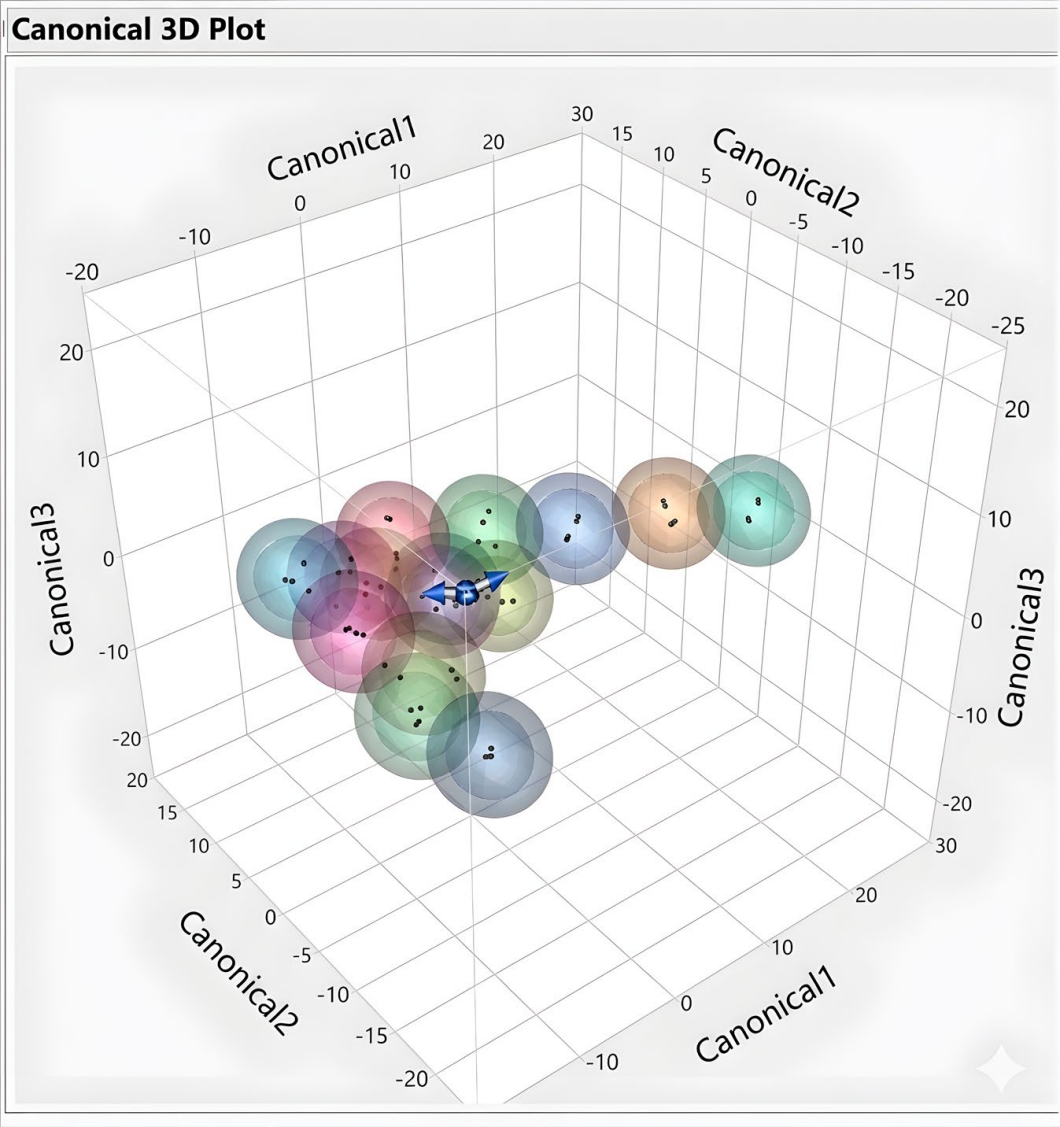
The 3D canonical plot of LDA results in the discrimination of 15 groups based on the first step of parameters, including malic acid, ascorbic acid, pH, and total sugar.

The discrimination of the 15 groups of adulterated apple juice concentrate with 4 replications was conducted based on malic acid, ascorbic acid, pH, and total sugar. The first, second, and third canonicals depicted 50.1%, 22%, and 19.1%, respectively, accounting for 91.2% of the total variation. Figure 3 appears as a 3D plot for the first three canonical obtained using LDA for 10% (batch 1), 20% (batch 2), 30% (batch 3), 40% (batch 4), and 50% (batch 5) glucose and fructose syrup, as well as 10% (batch 1), 20% (batch 2), 30% (batch 3), 40% (batch 4), and 50% (batch 5) date concentrate as adulterants for apple juice concentrate. The samples were recognized clearly based on the type of adulterants (GS, FS, and DC), and the predictive model had no misclassified samples. Thus, malic acid, ascorbic acid, pH, and total sugar as PCs in LDA, with a 3.61–2 log‐likelihood factor and 100% correct classification, showed the best discrimination for the type and concentration of the adulterants. For comparison, the combination of sugar and organic acid compounds provided a palatable classification of the 85 fruit juice samples, with a 100% correct classification rate for both the first and second cross‐validation methods. In this research, the data on the organic acid amount were utilized for LDA, resulting in a correct classification rate of 95.3% for the first and 94.1% for the cross‐validation method (Li et al. 2020). Another combination of diverse datasets (two or three of ordinary quality parameters, volatile compounds, and minerals) for LDA was performed in the separation of cherries according to the cultivar, achieving a more precise division of cultivars compared to those using an exclusive dataset of ordinary quality parameters, volatile compounds, or minerals (Papapetros et al. 2018).

#### 3.3.2 Second Step

**FIGURE 4:**
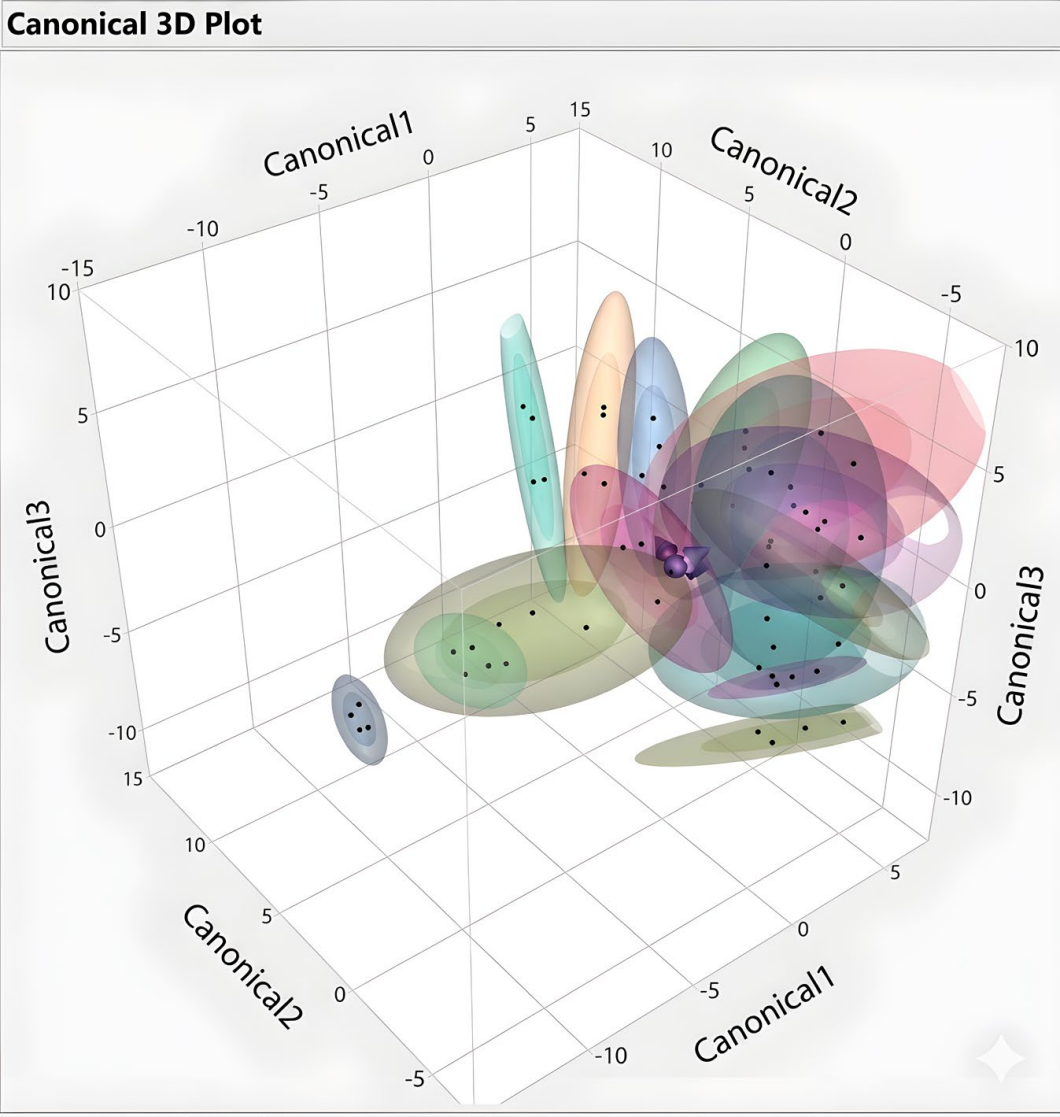
The 3D canonical plot of QDA results in the discrimination of 15 groups based on the second step of parameters, including reducing sugar, formalin index, and malic acid.

To demonstrate another effective method for separating the PAJC cluster from the adulterated samples, the number of variables used in each plot was decreased. The optimal separation between each adulterant and apple juice concentrate can be achieved by choosing only reducing sugar, formalin index, and malic acid, which are the best correlated with the adulterant and apple juice concentrate. The 3D plot in Figure 4 shows a significant improvement in separation using only three variables (reducing sugar, formalin index, and malic acid), with PC1, PC2, and PC3 now accounting for 95% of sample variation with a 9.11–2 log‐likelihood factor and 3 misclassifications.

#### 3.3.3 Third Step

**FIGURE 5:**
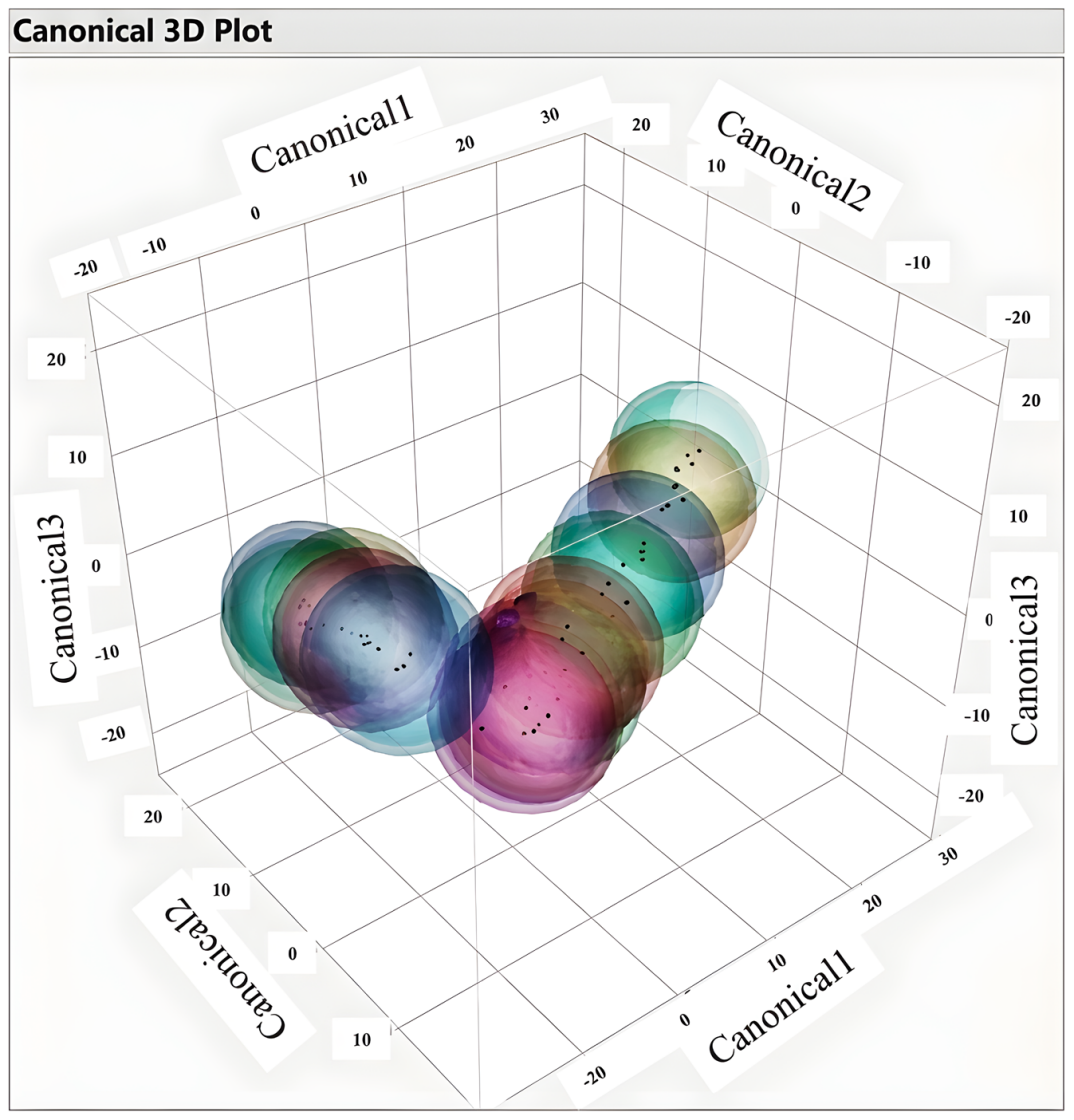
The 3D canonical plot of QDA results in the discrimination of 15 groups based on the third step of parameters, including ascorbic acid, malic acid, total sugar, pH, Cu, and K.

In this step, ascorbic acid, malic acid, total sugar, pH, Cu, and K were used to discriminate the 15 groups of AAJC with 4 replications. Figure 5 presents a canonical diagram in three dimensions of the first three canonical values acquired with QDA. The first three canonicals represented 100% of the total variance, and the precise categorization was 99% with a 3.1–2 log‐likelihood. The QDA by these mentioned factors for the discrimination of adulterated apple juice concentrate had no misclassification.

#### 3.3.4 Fourth Step

**FIGURE 6:**
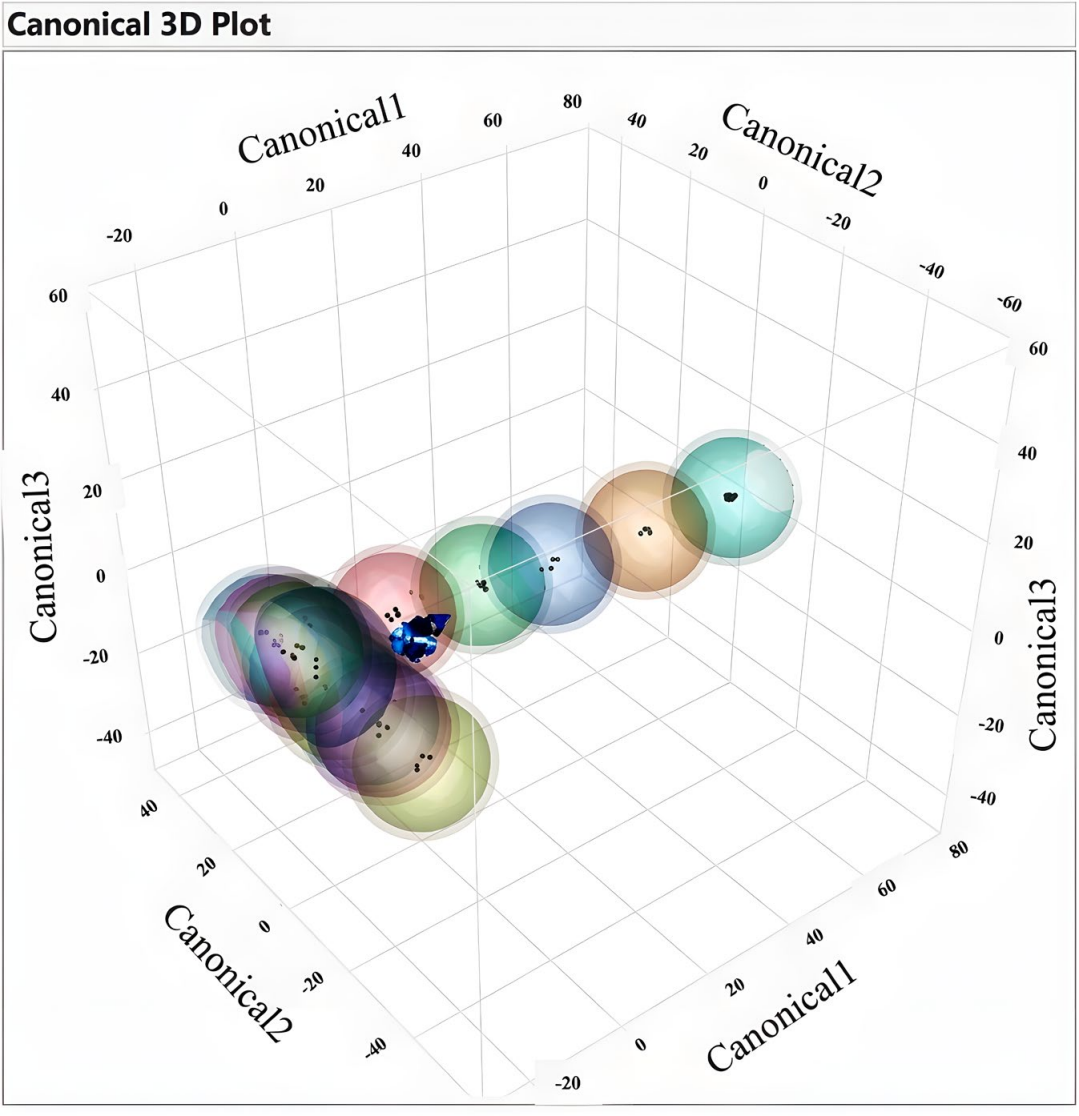
The 3D canonical plot of QDA results in the discrimination of 15 groups based on the fourth step of parameters, including pH, titrable acidity, formalin index, reducing sugar, total sugar, malic acid, ascorbic acid, lactic acid, fumaric acid, Mg, Fe, Zn, Cu, Na, and K.

The fourth step was obtained from a combination of all studied parameters, including pH, titratable acidity, formalin index, reducing sugar, total sugar, malic acid, ascorbic acid, lactic acid, fumaric acid, Mg, Fe, Zn, Cu, Na, and K responses of the 15 groups of adulterated apple juice concentrate with 4 replications, and utilized for discrimination by QDA. These parameters had a 100% accurate categorization with a 0.002–2 log‐likelihood and no misclassifications. Figure 6 indicates the 3D canonical schematic of the first three canonicals.

## 4 Conclusions

Despite the significant distinctions in the content of exclusive physicochemical properties, minerals, and organic acids profiles among apple juice concentrate, date concentrate, glucose syrup, and fructose syrup, the distribution of physicochemical properties, minerals, and organic acids in juices appeared to have a few distinct characteristics, which can facilitate the separation of natural apple juice concentrate and adulterated apple juice concentrate. This study conducted chemometric analysis to investigate the potential of physicochemical properties, minerals, and organic acids profiles of apple juice concentrate as PCs to distinguish and categorize adulteration with pure and mixed adulterants. A comparison of malic acid, ascorbic acid, pH, and total sugar in apple concentrate, in combination with LDA, was found to classify the apple concentrate according to the type and concentration of the adulterants. On the other hand, measurement of malic acid, formalin index, and reducing sugar in apple juice concentrate, in combination with QDA, yielded reliable results in identifying the type and concentration of the adulterants. Also, the study of ascorbic acid, malic acid, total sugar, pH, Cu, and K using QDA showed an accurate classification with no misclassifications. Lastly, we can suggest this type of application as a potential tool to assist the beverage industry and regulatory authorities in the field of food quality control, enabling the detection of fruit juice concentrate adulteration through quick and dependable screening analyses. Further investigation could achieve expanded classification strategies to identify apple juice concentrate samples adulterated with mixtures of diverse adulterants.

However, this study may have some limitations; for example, it focuses on apple juice concentrate, so the findings may not be directly applicable to other types of juices or food products. The reliance on specific analytical techniques (e.g., HPLC) might limit the generalizability of the methodology to laboratories without access to similar equipment. If the study uses a limited number of samples, the results might not be representative of the broader population of apple juice products, and the study may only cover a subset of possible adulterants, leaving some types of adulteration undetected.

## Author Contributions

**Saber Amiri:** data curation, visualization, writing – review and editing, writing – original draft. **Khalil Farhadi:** conceptualization, writing – original draft, writing – review and editing, supervision, methodology, project administration. **Samal Yeganeh‐Zare:** conceptualization, investigation, methodology, formal analysis, data curation, writing – original draft, writing – review and editing.

## Conflicts of Interest

The authors declare no conflicts of interest.

## Supporting information


**Figure S1:** Scheme of apple juice concentration formulation (total samples = 60 with 4 réplications for each batch).
**Figure S2:** Chromatogram from HPLC‐DAD showing organic acids profile with corresponding retention times in adulterated apple juice concentrated with 40% glucose syrup.
**Figure S3:** Chromatogram from HPLC‐DAD showing organic acids profile with corresponding retention times adulterated apple juice concentrated with 40% date concentrate.

## Data Availability

The data that support the findings of this study are available from the corresponding author upon reasonable request.
