# Supplementary material for: Detection of Adulterants in Apple Juice Concentrate by Physicochemical Properties, Organic Acids Profile, Minerals, and Multivariate Classification Strategies
Source: Food Sci Nutr. 2026 Jun 16;14(6):e72035. doi: 10.1002/fsn3.72035 (PMC13272632; doi:10.1002/fsn3.72035)
Supplement: Supplementary file 1 — Figure S1: Scheme of apple juice concentration formulation (total samples = 60 with 4 réplications for each batch). Figure S2: Chromatogram from HPLC‐DAD showing organic acids profile with corresponding retention times in adulterated apple juice concentrated with 40% glucose syrup. Figure S3: Chromatogram from HPLC‐DAD showing organic acids profile with corresponding retention times adulterated apple juice concentrated with 40% date concentrate. [file FSN3-14-e72035-s001.docx]

**Supplementary Materials**

**Detection of adulterants in apple juice concentrate by physicochemical properties, organic acids profile, minerals, and multivariate classification strategies**

**Samal Yeganeh-Zare, Khalil Farhadi, Saber Amiri**

**
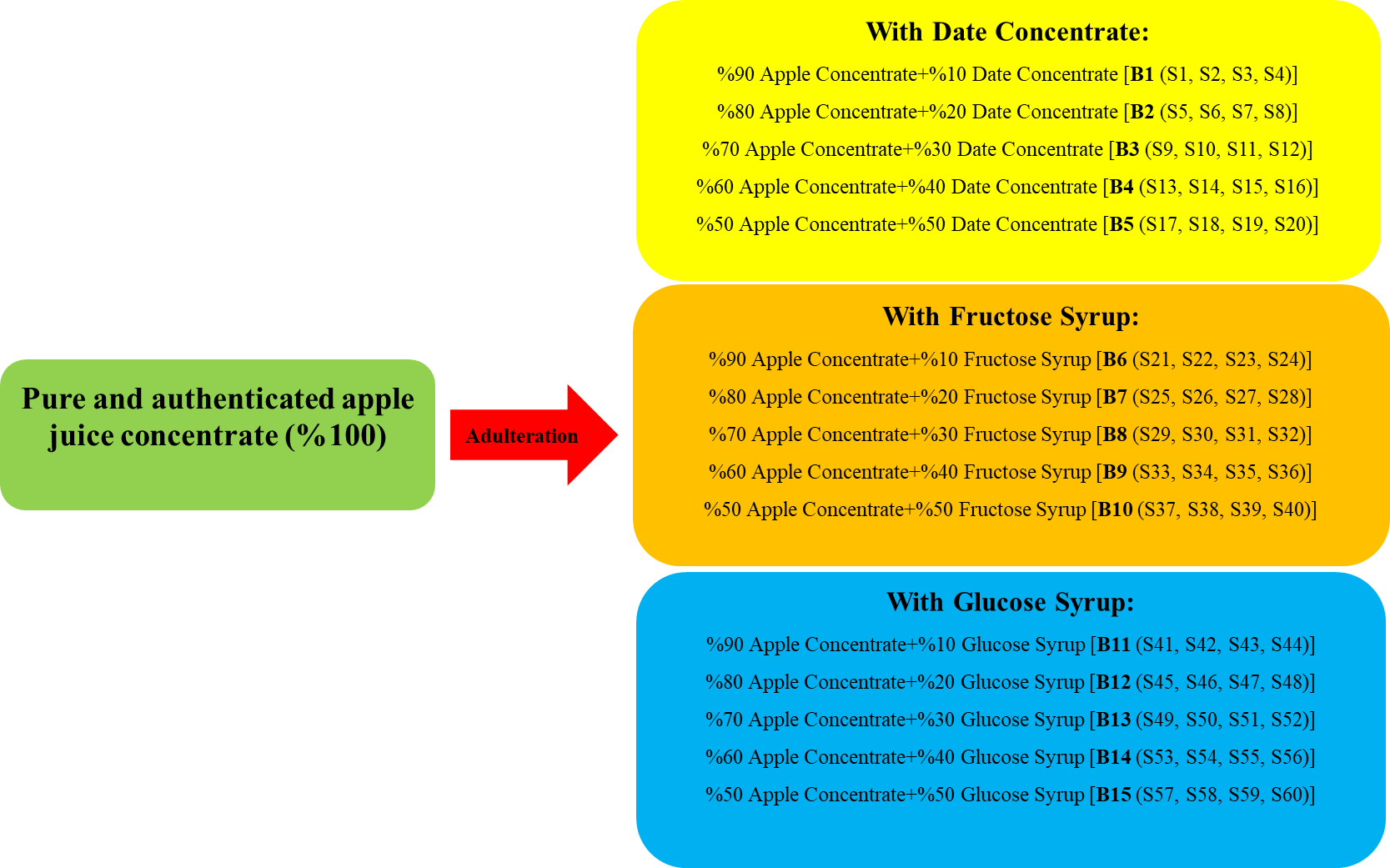
**

**Fig. S1:** Scheme of apple juice concentration formulation (total samples =60 with 4 réplications for each batch).


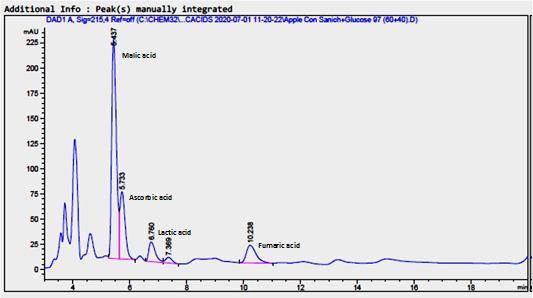


**Fig. S2:** Chromatogram from HPLC-DAD showing organic acids profile with corresponding retention times in adulterated apple juice concentrated with 40% glucose syrup


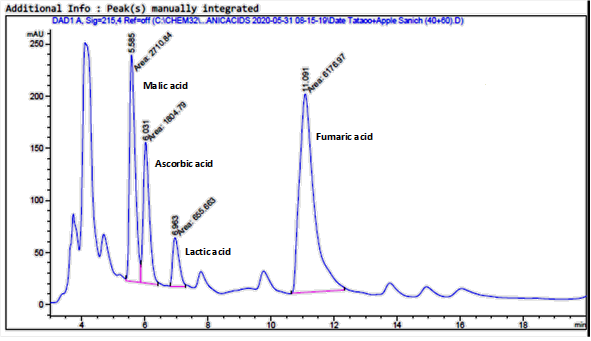


**Fig. S3:** Chromatogram from HPLC-DAD showing organic acids profile with corresponding retention times adulterated apple juice concentrated with 40% date concentrate
